# Society Meetings

**Published:** 1898-08

**Authors:** 


### SOCIETY MEETINGS.

THE American Association of Obstetricians and Gynecologists will hold its eleventh annual meeting in the banquet hall of the Monongahela House, at Pittsburg, Pa., Tuesday, Wednesday and Thursday, September 20, 21 and 22, 1898. The following papers have been promised :

1. ................., President's address, Charles A. L. Reed, Cincinnati.

2. ...................., Clinton Cushing, San Francisco.

3. Septic infection of ovarian cystoma, Charles Greene Cumston, Boston.

4. Recent experiences with the Alexander operation, H. E. Hayd, Buffalo.

5. Nursing in abdominal surgery, Joseph Price, Philadelphia.

6. Carcinoma of the breast, W. F. Westmoreland, Atlanta.

7. ................... D. Tod Gilliam, Columbus.

8. Organisation of major operations in private practice, W. G. Macdonald, Albany.

9. Explanation of the character of the temperature in appendicitis, Robert T. Morris, New York.

10. Pathological and clinical phases of gall-stone, A. H. Cordier, Kansas City.

11. Some facts in regard to uterine fibroids, H. D. Ingraham, Buffalo.

12. Albuminuria complicating gynecological operations, Rufus B. Hall, Cincinnati.

13. Extrauterine pregnancy with specimen; mature fetus borne twelve years, W. J. Asdale, Pittsburg.

14. Surgical treatment of morbid conditions involving the broad ligaments, A. P. Claike, Cambridge.

15. ..................., A. Vander Veer, Albany.

16. A second paper on the surgical treatment of intussusception in infants, with cases, H. Howitt, Guelph, Ont.

17. Relation of nervous affections to diseases of female pelvic organs, B. Sherwood Dunn, Boston.

18. Ureteral anastomosis, Geo. H. Noble, Atlanta.

19. ...................., W. H. Humiston, Cleveland.

20. Some of the complications following vaginal hystero-salpingo-oophorectomy in pelvic suppuration, F. Blume, Allegheny.

21. The question of intra-abdominal drainage, Edwin Walker, Evansville.

Correspondence pertaining to further contributions is pending. It is expected that this will be an unusually interesting meeting. One session will be set apart to the presentation of pathologic specimens and the consideration of their histories.

The Monongahela House will be the headquarters of the association and the management offers a reduction in rates to fellows and guests of the association. An invitation is extended to the members of the medical profession interested in the work of the association to attend the sessions.

---

The Niagara Falls Academy of Medicine held its regular meeting at the office of Dr. McCarty, Niagara avenue, Monday evening, July 11, 1898. A large number of members were present. The annual election of officers resulted as follows: President, T. J. McBlain; vice-president, W. J. Faulkner; secretary and treasurer, W. A. Scott; trustees, Owen McMarthy, F. McBrien, J. A. Miller. Papers were read by Dr. Bingham and Dr. McBlain, the latter on Care and treatment after abdominal operations.

---

The American Electro-therapeutic Association will hold its eighth annual meeting in the rooms of the Society of Natural Sciences, at Buffalo, September 13-15, 1898, under the presidency of Dr. Charles R. Dickson, of Toronto. The secretary is Dr. John Gerin, of Auburn, and the chairman of the committee of arrangements is Dr. Ernest Wende, of Buffalo. A large attendance is expected and a program of interest is arranging.

---

The State and Provincial Boards of Health of North America will hold their thirteen annual meeting at Detroit, August 9, 10 and 11, 1898, under the presidency of Dr. Benjamin Lee, of Philadelphia.

The first day, August 9th, will be given to meeting with the Michigan quarter. centennial celebration, in effecting organisation and in hearing reports of committees.

On the invitation of Governor Pingree and the health department, Dr. Ernest Wende, health commissioner of Buffalo, will read a paper on the Municipal prevention of disease.

---

THE Buffalo Academy of Medicine held meetings during the months of June and July, 1898, as follows :

*Section on Surgery*—Tuesday evening, June 7th, program : The use of nosophen and antinosine in disease of the middle ear, by Dr. F. H. Willmer ; Exhibition of case of fracture of skull, by Dr. Marshall Clinton ; Exhibition of specimens, by Drs. H. E. Hayd and C. P. Smith.

*Section on Pathology*—Tuesday evening, June 21st, program : Dorsokeratosis, with presentation of patient, etc., by Dr. Grover W. Wende ; Myxedema, with presentation of a case, by Dr. J. C. Clemesha ; A pathologic exhibit, by Dr. Charles E. Congdon.

*Section on Obstetrics*—Tuesday evening, June 28th, program : Discussion and diagnosis of suppurative forms of intrapelvic disease and its surgical management, by Dr. Joseph Price, of Philadelphia.

A special meeting of the Academy was held Monday evening, July 11, program : The production of diastase and of an alcoholic ferment from fungi, by Dr. Jokichi Takamine, formerly of Tokyo University, Tokyo, Japan.

The annual meeting of the Academy was held Tuesday, June 14, 1898.

The following is the roster of officers, for the session of 1898-1899 : President, Dr. Roswell Park ; secretary, Dr. Thomas F. Dwyer ; treasurer, Dr. Charles S. Jewett ; trustees, Drs. B. G. Long (3 years), M. Hartwig (2 years), DeLancey Rochester (1 year). Section on medicine : chairman, Dr. William G. Ring ; secretary, Dr. Fridolin Thoma. Section on surgery : chairman, Dr. John Parmenter ; secretary, Dr. J. Henry Dowd. Section on obstetrics : chairman, Dr. William G. Taylor ; secretary, Dr. N. Gorham Russell. Section on pathology : chairman, Dr. A. L. Benedict , secretary, Dr. A. G. Bennett. Council : the above officers and the president of the academy of the previous year, Dr. Lucien Howe.

